# HPV18 E6 inhibits α‐ketoglutarate‐induced pyroptosis of esophageal squamous cell carcinoma cells via the P53/MDH1/ROS/GSDMC pathway

**DOI:** 10.1002/2211-5463.13666

**Published:** 2023-07-08

**Authors:** Duo Tang, Yuchen Zheng, Guozhen Wang, Chao Sheng, Zijia Liu, Biqi Wang, Qi Zong, Yuchen Zhang, Xiaonan Hou, Mengfei Yao, Zhixiang Zhou

**Affiliations:** ^1^ Beijing International Science and Technology Cooperation Base of Antivirus Drug, Department of Biology, Faculty of Environment and Life Beijing University of Technology Beijing China; ^2^ Department of Clinical Laboratory China‐Japan Friendship Hospital Beijing China

**Keywords:** E6 oncogene, GSDMC, HPV, pyroptosis, ROS, α‐ketoglutarate

## Abstract

Oncogene E6 plays a critical role in the development and progression of esophageal cancer caused by human papillomavirus (HPV) infection. Alpha‐ketoglutarate (AKG) is a key metabolite in the tricarboxylic acid cycle and has been widely used as a dietary and anti‐ageing supplement. In this study, we found that treating esophageal squamous carcinoma cells with a high dose of AKG can induce cell pyroptosis. Furthermore, our research confirms that HPV18 E6 inhibits AKG‐induced pyroptosis of esophageal squamous carcinoma cells by lowering P53 expression. P53 downregulates malate dehydrogenase 1 (MDH1) expression; however, MDH1 downregulates L‐2‐hydroxyglutarate (L‐2HG) expression, which inhibits a rise in reactive oxygen species (ROS) levels—as L‐2HG is responsible for excessive ROS. This study reveals the actuating mechanism behind cell pyroptosis of esophageal squamous carcinoma cells induced by high concentrations of AKG, and we posit the molecular pathway via which the HPV E6 oncoprotein inhibits cell pyroptosis.

## Graphical abstract

This research found that α‐ketoglutarate (AKG) can cause pyroptosis of esophageal squamous cell carcinoma cells. AKG‐induced pyroptosis is caused by reactive oxygen species (ROS), produced during the conversion of AKG to L‐2HG. The presence of human papillomavirus (HPV) significantly inhibits AKG‐induced pyroptosis, which was mainly attributed to the inhibition of pyroptosis by HPV E6 via the p53/MHD1/ROS/GSDMC pathway.

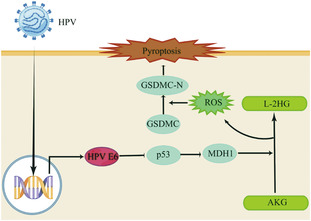

**Abbreviations**
AKG: alpha‐ketoglutarate
BMI: body mass index
DM‐AKG: dimethyl‐α‐ketoglutaric acid
DMEM: Dulbecco's modified Eagle medium
DR6: death receptor 6
EC: esophageal cancer
ESCC: esophageal squamous cell carcinoma
GAPDH: glyceraldehyde 3‐phosphate dehydrogenase
GSDMA: gasdermin A
GSDMB: gasdermin B
GSDMC: gasdermin C
GSDMD: gasdermin D
GSDME: gasdermin E
GZMA: granzyme A
HPV: human papillomavirus
L‐2HG: L‐2‐hydroxyglutarate
LDH: lactate dehydrogenase
MDH1: malate dehydrogenase 1
PJVK: Pejvakin
ROS: reactive oxygen species
SD: standard deviation
TCA: tricarboxylic acid

Pyroptosis is a highly proinflammatory programmed cell death that is intricately linked to inflammation [1]. Inflammatory corpuscles are activated when cells eliminate pathogens or damaging factors that have invaded the body, which in turn induces the activation of the caspase family. The activated caspase initiates the activation of the caspase‐dependent gasdermin family of proteins and the maturation and release of inflammatory factors IL‐1β and IL‐18 [2]. The gasdermin family is a significant executor of pyroptosis and comprises Gasdermin A (GSDMA), Gasdermin B (GSDMB), Gasdermin C (GSDMC), Gasdermin D (GSDMD), Gasdermin E (GSDME, also known as DFNA5) and Pejvakin (PJVK, also known as DFNB59) [3]. All members of the gasdermin family, excluding PJVK, have two domains: a self‐inhibition domain, the C segment, and a functional domain, the N segment [4]. The entire length of a gasdermin protein is not involved in inducing pyroptosis. However, when the full‐length protein is cut to remove the C‐terminal domain, the N‐terminal domain perforates the cell membrane to induce pyroptosis [3]. The roles of most members of the gasdermin family in pyroptosis have been proven. Group A Streptococcus, a human pathogen, secretes a protease virulence factor, SpeB, which cleaves GSDMA, inducing GSDMA‐dependent pyroptosis [5, 6]. GSDMB is cleaved by granzyme A (GZMA) in toxic lymphocytes, thus inducing pyroptosis; GSDMB is also cleaved by Caspase‐1/4 in cells, which induces pyroptosis [7, 8, 9]. GSDMC is cut by Caspase‐8, removing its N terminus and thus inducing pyroptosis [10]. Caspase‐1 and Caspase‐4/5/11 specifically cleave the linker between the amino‐terminal gasdermin‐N domain and the carboxyl‐terminal gasdermin‐C domain in GSDMD, releasing the GSDMD‐N domain to induce pyroptosis [11, 12]. When GSDME expression in cells is sufficient, Caspase 3 cuts GSDME, switching the cell death mode from apoptosis to pyroptosis [13, 14]. The mechanism underpinning the role of PJVK in pyroptosis has not been reported, but PJVK is closely linked to nonsyndromic hearing impairment [15, 16].

Alpha‐ketoglutarate (AKG) is a critical intermediate of the tricarboxylic acid (TCA) cycle, and it plays a crucial role in many metabolic processes [17]. In the TCA cycle, AKG is converted into succinyl‐coenzyme A by α‐ketoglutarate dehydrogenase (OGDH), facilitating the fourth step in the TCA cycle. AKG also plays a significant role in nitrogen scavenging and amino acid metabolism. AKG can be converted into glutamic acid by glutamate dehydrogenase, and the glutamic acid obtained can be further metabolised by glutamine synthase into glutamine [18]. In day‐to‐day life, AKG is immensely significant as a metabolic intermediate. For example, AKG is a dietary supplement because it plays a specific role in anti‐ageing processes and body improvement. Furthermore, some studies have shown that AKG can improve hyperglycaemia symptoms in diabetic patients [19, 20, 21, 22, 23]. AKG has also been studied in antitumour research. For example, administering AKG combined with targeting BCAT1 can trigger the metabotropic synthetic death of glioblastoma [24] However, there are no published studies on AKG in antitumour research on esophageal cancer (EC). Some studies have reported the lethal cytotoxic effect of AKG; the cytotoxicity induced by AKG is most significant in cells with mitochondrial dysfunction [25, 26]. Regarding pyroptosis, AKG can induce pyroptosis by activating Caspase 8, which cuts GSDMC via death receptor 6 (DR6) [27]. In summary, AKG may be a double‐edged sword in the body, which makes investigating the toxic effects and mechanisms of AKG an interesting research focus.

Esophageal cancer is the seventh most common cancer in the world [28], and squamous cell carcinoma accounts for 90% of EC cases, especially in China, Central Asia, East Africa and South Africa [29]. A large body of evidence shows that human papillomavirus (HPV) is implicated in EC [30, 31]. HPV is a double‐stranded DNA virus that readily infects mucosal epithelial tissues in the anal genitalia and the upper respiratory and digestive tracts [32]. HPV infection is the leading cause of cervical cancer, and almost all cervical cancers are the result of HPV infection [33]. There is growing evidence that HPV is also associated with the development of vaginal, penile, anal, head and neck cancers [32]. The carcinogenic mechanism of HPV in EC is unclear. However, it is generally surmised that the products of the expression of the HPV E6 and E7 genes act on anticancer genes: the E6 protein degrades p53, and the E7 protein acts on pRb; consequently, the degradation and deactivation of proteins related to tumour suppressor genes promotes cell cycle disorder and engenders malignant proliferation of cells [34, 35]. A considerable number of studies have demonstrated that HPV infection can inhibit cell death and promote the development of malignant cancer. For example, HPV E6 degrades P53, thus engendering cell proliferation and inhibiting apoptosis, and E6 and E7 continuously impair autophagy by inhibiting the fusion of autophagosomes and lysosomes [36, 37, 38, 39]. There are only a few published studies on the relationship between HPV and pyroptosis. Only one study reports that HPV E7 promotes the degradation and ubiquitination of IFI16 inflammatory corpuscles, thus inhibiting pyroptosis induced by dsDNA transfection [40]. There are no published studies on whether HPV affects members of the gasdermin family and the mechanisms relevant to its impact.

We treated a variety of esophageal and cervical cancer cells with varying concentrations of AKG and found that high concentrations of AKG induce significant cytotoxicity and pyroptosis, and HPV‐positive cells were only slightly sensitive to AKG toxicity. With further experimental investigation, we found that HPV E6 inhibits AKG‐induced pyroptosis of esophageal squamous cell carcinoma (ESCC) cells, which is executed by p53, malate dehydrogenase 1 (MDH1), reactive oxygen species (ROS) or GSMDC.

## Materials and methods

### Cell culture and transfection

Five ESCC cell lines (KYSE150, KYSE180, KYSE450, EC109 and EC9706) and three cervical carcinoma cell lines (C33a, Siha and Hela) were cultured in Dulbecco's modified Eagle medium (DMEM; GIBCO, Grand Island, NY, USA), with 10% fetal bovine serum (GIBCO), 100 U·mL^−1^ penicillin and 100 mg·mL^−1^ streptomycin (Hyclone, Logan, UT, USA). The culture was then placed in a 5% CO_2_ constant temperature cell culture incubator (Thermo Scientific, Wilmington, DE, USA) at 37 °C. The KYSE150 cells were purchased from the Cell Bank of the Chinese Academy of Sciences, Shanghai, China. The KYSE450, EC109, EC9706, C33a, Siha and Hela cells were obtained from the Chinese Center for Disease Control and Prevention, Beijing, China. KYSE180 cells were obtained from Shantou University Medical College, Guangdong, China. KYSE150, KYSE180, KYSE450 and C33a cells are HPV‐negative cell lines, and EC109, EC9706, Siha and Hela cells are HPV‐positive cell lines [41, 42, 43, 44]. The HPV18 E6, E7 and p53 genes were obtained from the National Center for Biotechnology Information (NCBI) Gene database and synthesised by Tsingke Biotechnology Co., Ltd., Beijing, China (Fig. S1). The HPV18 E6, E7 and p53 genes were each assembled into a pcDNA3.1 vector, labelled pcDNA‐E6, pcDNA‐E7 and pcDNA‐P53, respectively. Synthesised siRNA‐MDH1 GCUGUUAGUGUGCAUUCUA, siRNA‐p53 GCAUGAACCGGAGGCCCAU and siRNA‐GSDMC CCUAGAAACUGUUGUGACA (Tsingke Biotechnology Co., Ltd., Beijing, China) were used to knock down MDH1, P53 and GSDMC expression levels. Plasmids and siRNA were transfected into cells using the jetPRIME transfection reagent (Polyplus‐transfection, Illkirch‐Graffenstaden, France). The siRNA‐N and pcDNA3.1 vectors comprised the control group.

### RNA extraction and real‐time quantitative PCR

PCR primers can be found at PrimerBank or designed using the oligo7 software (Molecular Biology Insights, Inc., Springs, CO, USA) by following the instructions in the software (Table 1). Total RNA was extracted from the cells using TRIzol reagent (Invitrogen, Carlsbad, CA, USA). A PrimeScript RT kit with gDNA Eraser (Takara, Dalian, China) was used to synthesise 1 μg of total RNA, with a final volume of 20 μL, for first‐strand cDNA synthesis. An SYBR Premix Dimer Eraser kit (TaKaRa Biotechnology, Dalian, China) and a ViiA7 real‐time quantitative PCR instrument were used to detect gene expression. Glyceraldehyde 3‐phosphate dehydrogenase (GAPDH) and β‐actin were used as internal controls. Four sets of each gene were repeated for a 10 μL PCR reaction. Predenaturation was performed at 95 °C for 30 s and then for 40 cycles—the parameters for each cycle were as follows: 5 s at 95 °C, 30 s at 55 °C and 30 s at 72 °C. The parameters for the melting curve cycle were as follows: 0 s at 95 °C, 15 s at 65 °C and 0 s at 95 °C. The $2^{-\Delta \Delta C_{T}}$ method was used to calculate relative gene expression levels.

**Table 1:** Primers of RT‐qPCR.

Primer	Primer sequence (5′–3′)
MDH1‐F	GGTGCAGCCTTAGATAAATACGC
MDH1‐R	AGTCAAGCAACTGAAGTTCTCC
P53‐F	CAGCACATGACGGAGGTTGT
P53‐R	TCATCCAAATACTCCACACGC
GSDMC‐F	TCCATGTTGGAACGCATTAGC
GSDMC‐R	CAAACTGACGTAATTTGGTGGC
GAPDH‐F	TGTTGCCATCAATGACCCCTTC
GAPDH‐R	AGCATCGCCCCACTTGATTTTG
B‐Actin‐F	CATGTACGTTGCTATCCAGGC
B‐Actin‐R	CTCCTTAATGTCACGCACGAT

### Reactive oxygen species detection

2 × 10^4^ cells were seeded into a 96‐well plate; an ROS inducer, Rosup (Beyotime, Nantong, China; Cat. # S0033S‐2), was used as the positive control, and *N*‐acetylcysteine (NAC; MedChenExpress, Princeton, NJ, USA; Cat. # HY‐B0215) was used as an ROS inhibitor. The NAC was incubated 24 h ahead of schedule. After 2 h of DM‐AKG (Shanghai Yuanye Bio‐Technology, Shanghai, China; Cat. # S30041) administration, the serum‐free medium containing 0.1% 2′,7′‐dichlorofluorescein diacetate (DCFH‐DA; Beyotime, Nantong, China; Cat. # S0033S‐1) was replaced. Following incubation at 37 °C for 20 min, the cells were washed three times with the serum‐free medium and were then screened and analysed with Operetta (PerkinElmer, Waltham, MA, USA), a high content profiler.

### Lactate dehydrogenase assay

Following the manufacturer's instructions, a lactate dehydrogenase (LDH) assay kit (Solarbio, Beijing, China; Cat. # BC0680) was used to detect the amount of LDH released by cells. The LDH absorbance was measured at 450 nm using a microplate reader (PerkinElmer). The experiment was independently repeated three times.

### Cell counting kit‐8 assay

2 × 10^4^ cells were seeded into a 96‐well plate to which a 90 μL medium and a 10 μL cell counting kit‐8 reagent (Dojindo, Kumamoto, Japan) were added, followed by incubation at 37 °C for 1 h. A microplate reader (PerkinElmer) was adopted to measure the absorbance (*A*) at 450 nm. The cell survival rate was calculated using the following formula: cell survival rate (%) = (*A*_the experimental group_/*A*_the control group_) × 100%. The experiment was independently repeated three times.

### L‐2HG content detection

Following the manufacturer's instructions, the cells were treated with the reagents in a Solarbio kit (Solarbio; Cat. # BC4704). L‐2‐hydroxyglutarate (L‐2HG, Cat. # 1038657) standard products purchased from Leyan (Shanghai, China). The L‐2HG concentration in the samples was measured using HPLC with an Agilent 1200 diode array detector (Agilent Corp., Santa Clara, CA, USA). The operating parameters for the HPLC determination were as follows: a C18 reversed‐phase column of 5 μm × 4.6 mm × 250 mm, a column temperature of 30 °C, a detection wavelength of 220 nm; and for the mobile phase: acetonitrile/ultrapure water (v/v, 70 : 30), an injection volume of 10 μL, and a flow rate of 0.4 mL·min^−1^. The relative L‐2HG levels were calculated using the following formula: peak area_the experimental group_/peak area_the control group_. Each sample was repeated independently three times.

### Western blot

Each group of cells was washed twice with ice‐cold PBS, and a radioimmunoprecipitation assay lysis buffer (Cell Signaling Technology, Danvers, MA, USA) containing a protease inhibitor mixture (Roche, Basel, Switzerland) was used for processing. The sample was centrifuged at a rate of 12 000 ***g*** for 10 min at 4 °C, and the supernatant was extracted. A bicinchoninic acid protein assay kit (Solarbio) was used to measure the total protein concentration in the sample, and the sample was diluted with PBS to make the concentration consistent. A 5× loading buffer (Applygen Technologies Inc., Beijing, China) was then added, and the mixture was boiled in a water bath at 100 °C for 5 min. The protein sample was processed using 10% of SDS/PAGE, a semidry method was used to transfer it to a polyvinylidene difluoride membrane (Millipore, Bedford, MA, USA), and 5% skimmed milk powder diluted with TBST was applied for 1 h for sealing. The membrane with the diluted primary antibody was incubated overnight. The primary antibodies included rabbit polyclonal antibody against GAPDH (1 : 5000; ProteinTech, Rosemont, IL, USA; Cat. # 10494‐1‐AP), rabbit polyclonal antibody against MDH1 (1 : 1000; ProteinTech; Cat. # 15904‐1‐AP), rabbit polyclonal antibody against p53 (1 : 1000; ProteinTech; Cat. # 10442‐1‐AP), rabbit polyclonal antibody against V5‐tag (1 : 1000; ProteinTech; Cat. # 14440‐1‐AP), rabbit polyclonal antibody against GSDMB (1 : 1000; Abcam, Cambridge, UK; Cat. # ab215729), rabbit polyclonal antibody against GSDMC (1 : 1000; Abcam; Cat. # ab225635), rabbit polyclonal antibody against GSDMD (1 : 1000; Abcam; Cat. # ab209845), rabbit polyclonal antibody against GSDME (1 : 1000; Abcam; Cat. # 215191) and rabbit polyclonal antibody against caspase‐8 (1 : 1000; ProteinTech; Cat. # 13423‐1‐AP). The membrane was then washed three times with TBST for 10 min each time. Next, with secondary antibody (KPL) was incubated with marked Dylight 680 for 1 h. Finally, an Odyssey infrared imaging system (LI‐COR Biosciences, Lincoln, NE, USA) was used for screening, and quantification was performed using the imagej software (National Institutes of Health, Bethesda, MD, USA).

### Statistical analysis and graphing

All data graphs were constructed using the graphpad prism 7.04 software (GraphPad Software, San Diego, CA, USA). All statistical analyses were performed using graphpad prism 7.04 and the Statistical Package for the Social Sciences (spss; IBM Corp., Armonk, NY, USA). The results of the data analyses are expressed as the mean ± standard deviation (SD). Comparisons between two groups were made using an unpaired *t*‐test, while comparisons between multiple groups were made using a one‐way analysis of variance (ANOVA). *P* < 0.05 was considered significant. Graphic abstract drawing by figraw (www.figdraw.com, authorisation ID: TRUYT8ff28).

## Results

### AKG induces pyroptosis of ESCC cells

**Fig. 1:**
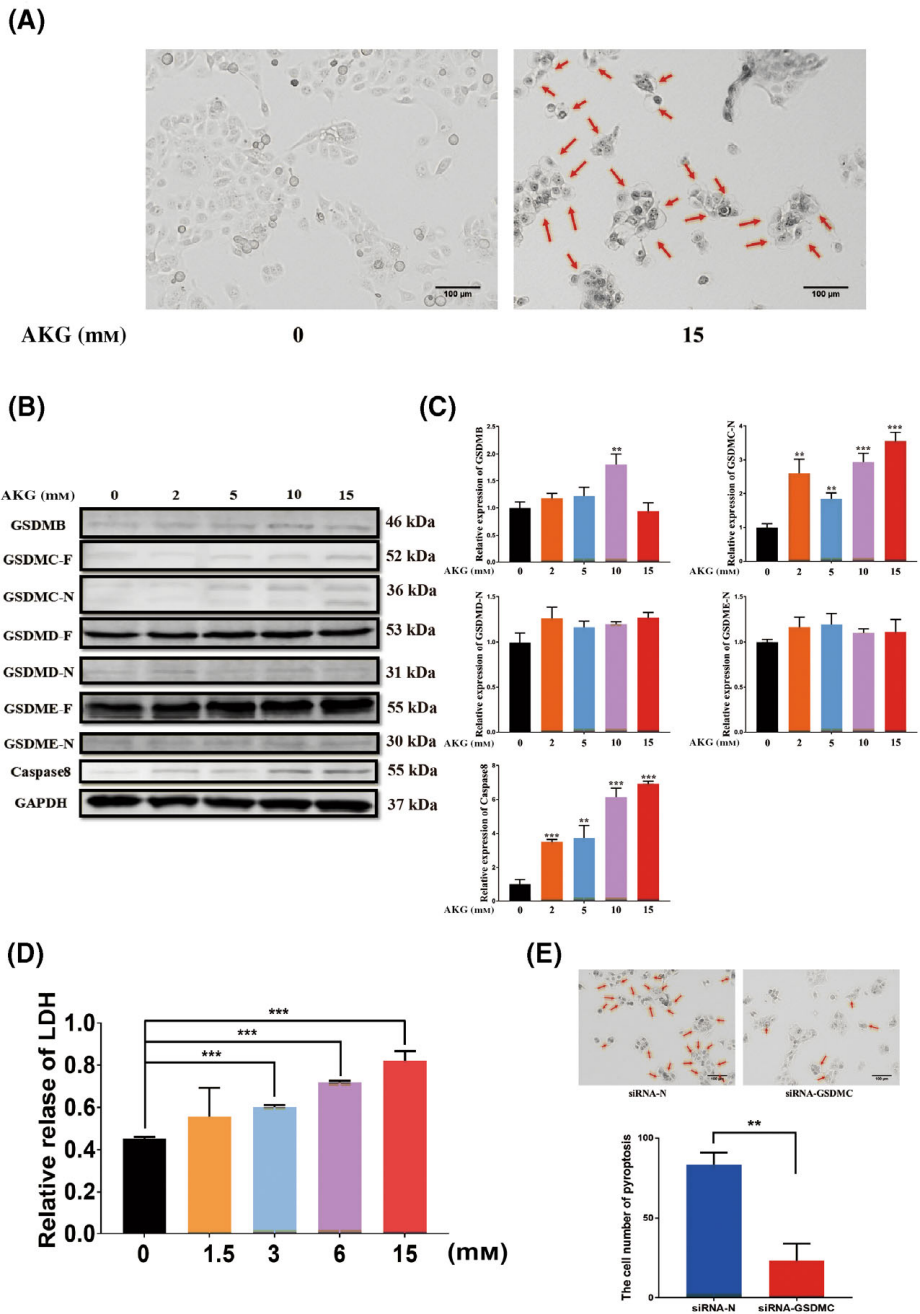
Alpha‐ketoglutarate induces the pyroptosis of ESCC cells. (A) Image observation of pyroptosis of KYSE150 cells induced by DM‐AKG, the 0 mm group was supplemented with DMSO without DM‐AKG in the same amount, scale bar = 100 μm. (B, C) After 10 h of exposure to DM‐AKG, expression of GSDMB, GSDMC, GSDMD, GSDME and caspase‐8 detected via western blotting, data were analysed using one‐way ANOVA with Tukey's *post hoc* test, **P < 0.01, ***P < 0.001. (D) Relative levels of LDH released after adding varying concentrations of DM‐AKG, data represent the mean ± SD of four independent experiments, data were analysed using one‐way ANOVA with Tukey's *post hoc* test, ***P < 0.001. (E) Image observation and pyroptosis statistics after knocking down GSDMC, scale bar = 100 μm, data represent the mean ± SD from three random views, data were analysed using Student's *t*‐test, **P < 0.01.

Because AKG cannot cross the cell membrane, dimethyl‐α‐ketoglutaric acid (DM‐AKG) was used. After entering the cell membrane, DM‐AKG was hydrolysed into AKG [45]. When the ESCC cell KYSE150 was treated with a high concentration of DM‐AKG, we found that a high concentration of DM‐AKG‐induced cell death, with the characteristics of pyroptosis, that is the cells swelled, rounded and blistered (Fig. S2, Fig. 1A, Movie S1). To exclude the potential toxicity of DM modified compounds themselves. We used DM‐succinate as a control treatment, and the results confirmed that the toxicity was specific to AKG at concentrations of 15 and 30 mm and was not caused by DM‐modified compounds (Fig. S3). We believe that the toxic effect of AKG‐induced cell death in ESCC cells is accompanied by pyroptosis. To determine how AKG induces pyroptosis in ESCC cells, we screened for the expression of GSDMB, GSDMC, GSDMD and GSDME, which are the key pyroptosis execution proteins. Because cells exposed to high concentrations of DM‐AKG could not extract proteins after 12 h, we chose the tenth hour after cell exposure to DM‐AKG for protein extraction and detection. We found that AKG‐induced pyroptosis is executed by GSDMC and not GSDMB, GSDMD or GSDME (Fig. 1B). The expression levels of GSDMC‐N and caspase‐8 increased with an increase in the AKG concentration, and caspase‐8 was the key upstream activator of GSDMC (Fig. 1C). Concurrently, with an increase in DM‐AKG concentration, there was also a significant increase in the levels of LDH released by the cells (Fig. 1D). Subsequently, GSDMC was knocked down by siRNA, and the pyroptosis phenotype of the cells became apparent (Fig. 1E, Fig. S4A). This clearly shows that AKG induces pyroptosis of ESCC cells, which is executed by GSDMC.

### AKG‐induced pyroptosis is caused by ROS

**Fig. 2:**
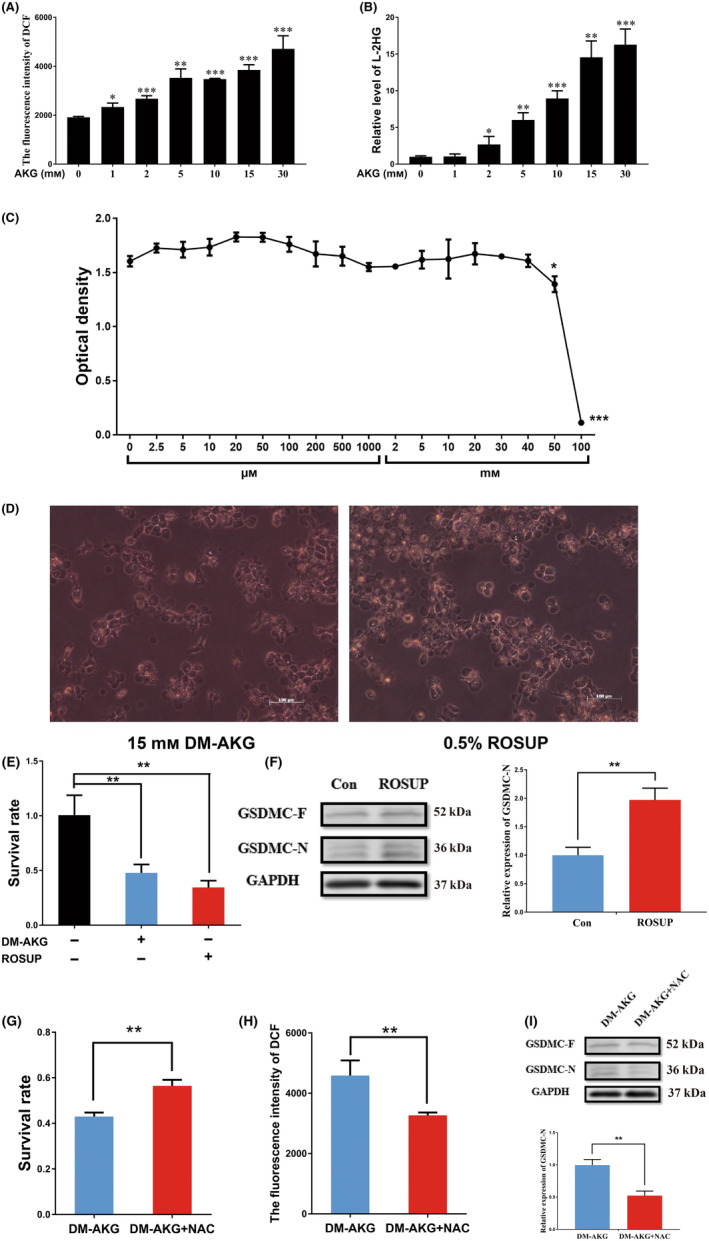
Alpha‐ketoglutarate‐induced pyroptosis caused by ROS. (A) After 6 h of exposure to DM‐AKG, ROS produced by different concentrations of DM‐AKG, data represent the mean ± SD of four independent experiments, data were analysed using one‐way ANOVA with Tukey's *post hoc* test, *P < 0.05, **P < 0.01, ***P < 0.001. (B) After 6 h of exposure to DM‐AKG, detected L‐2HG produced by DM‐AKG at different concentrations, data represent the mean ± SD of three independent experiments, data were analysed using one‐way ANOVA with Tukey's *post hoc* test, *P < 0.05, **P < 0.01, ***P < 0.001. (C) Toxic effect of different concentrations of DM‐L‐2HG, data represent the mean ± SD of three independent experiments, data were analysed using one‐way ANOVA with Tukey's *post hoc* test, *P < 0.05, ***P < 0.001. (D) Image observation of Rosup‐induced cell death and DM‐AKG induced cell death, scale bar = 100 μm. (E) Cell survival rate after exposure to Rosup and DM‐AKG 24 h, data represent the mean ± SD of four independent experiments, data were analysed using one‐way ANOVA with Tukey's *post hoc* test, **P < 0.01. (F) After 10 h of exposure to Rosup, western blot detection of Rosup‐induced GSDMC expression, data were analysed using Student's *t*‐test, **P < 0.01. (G) Cell survival rate of ESCC cells after NAC treatment, data represent the mean ± SD of four independent experiments, data were analysed using Student's *t*‐test, **P < 0.01. (H) ROS production in ESCC cells after NAC treatment, data represent the mean ± SD of four independent experiments, data were analysed using Student's *t*‐test, **P < 0.01. (I) GSDMC expression detected via western blotting after NAC treatment, data were analysed using Student's *t*‐test, **P < 0.01.

When the ESCC cells were treated with DM‐AKG, we found that the ESCC cells also produced a large amount of ROS, which was positively correlated with the concentration of DM‐AKG (Fig. 2A). Studies have shown that AKG can be catalysed by MDH1 to produce L‐2HG, and thus, a large amount of ROS [27, 46, 47]. When we screened for L‐2HG, we also found that the L‐2HG levels were positively correlated with the concentration of DM‐AKG (Fig. 2B). These results confirm that AKG produced L‐2HG, thus releasing a large amount of ROS upon entering the cells. Subsequently, we investigated whether ROS and L‐2HG have an impact on defocus. Because L‐2HG cannot cross the cell membrane, dimethyl‐L‐2HG (DM‐L‐2HG) was used. When ESCC cells were treated with various concentrations of DM ‐L‐2HG, significant toxicity was observed only when the concentration of DM ‐L‐2HG exceeded 50 mm (Fig. 2C). After using an ROS‐positive agent, Rosup, the cell phenotype of the Rosup group of cells was the same as that of the AKG group, and cytotoxicity was produced (Fig. 2D,E). The levels of GSDMC‐N in the Rosup group were high (Fig. 2F). These results indicate that ROS does indeed cause an increase in GSDMC‐N expression, thus inducing pyroptosis. After treating the cells in the AKG group with NAC, an ROS inhibitor, the ROS levels were significantly inhibited, GSDMC‐N production decreased and the cell survival rate improved (Fig. 2G–I). These results fully confirm that the ROS produced after DM‐AKG treatment cause pyroptosis, not L‐2HG.

### HPV E6 inhibits AKG‐induced pyroptosis

**Fig. 3:**
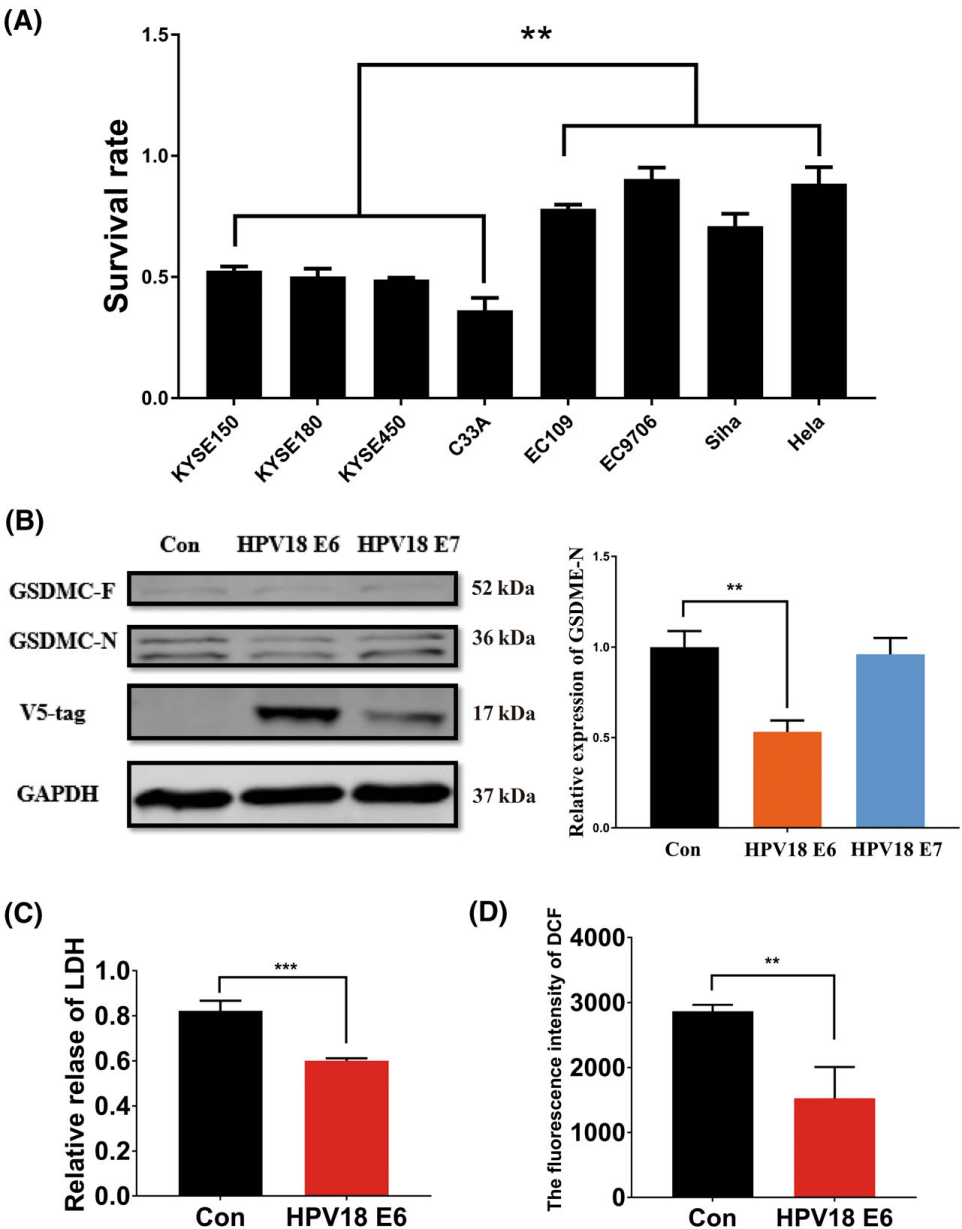
Human papillomavirus E6 inhibits AKG‐induced pyroptosis. (A) Comparison of the survival rates of HPV‐negative cells and HPV‐positive cells at a DM‐AKG concentration of 15 mm after 24 h, data represent the mean ± SD of four independent experiments, data were analysed using Student's *t*‐test, **P < 0.01. (B) Effect of HPV E6 and E7 on GSDMC‐N detected via western blotting at a DM‐AKG concentration of 15 mm after 10 h, data were analysed using one‐way ANOVA with Tukey's *post hoc* test, **P < 0.01. (C) Relative levels of LDH released following HPV E6 expression, data represent the mean ± SD of four independent experiments, data were analysed using Student's *t*‐test, ***P < 0.001. (D) Relative levels of ROS released following HPV E6 expression at a DM‐AKG concentration of 15 mm, data represent the mean ± SD of four independent experiments, data were analysed using Student's *t*‐test, **P < 0.01.

We observed something interesting while using DM‐AKG to induce cytotoxicity: the resistance of HPV‐positive cells (EC109, EC9706, Siha and Hela) to DM‐AKG at 15 mm was significantly higher than the resistance of HPV‐negative cells (KYSE150, KYSE180, KYSE450 and C33a) (Fig. 3A). This result could be related to the carcinogenic characteristics of HPV. Because the carcinogenic function of HPV is realised primarily by HPV E6 and HPV E7. Therefore, we constructed HPV E6‐ and E7‐positive cells from KYSE150 cells to investigate the effect of HPV E6 or E7 on pyroptosis. We added a V5‐tag to E6 and E7 to detect the expression of the oncoproteins. HPV E6 expression significantly inhibited the production of GSDMC‐N (Fig. 3B). After pcDNA‐E6 was transferred into KYSE150 and C33A cells, DM‐AKG‐induced cytotoxicity was significantly inhibited (Fig. S5). Concurrently, HPV E6 expression significantly inhibited the release of LDH and the production of ROS (Fig. 3C,D). These results confirm that HPV E6 inhibits DM‐AKG‐induced pyroptosis.

### HPV E6 inhibits pyroptosis via the p53/MDH1/ROS/GSDMC pathway

**Fig. 4:**
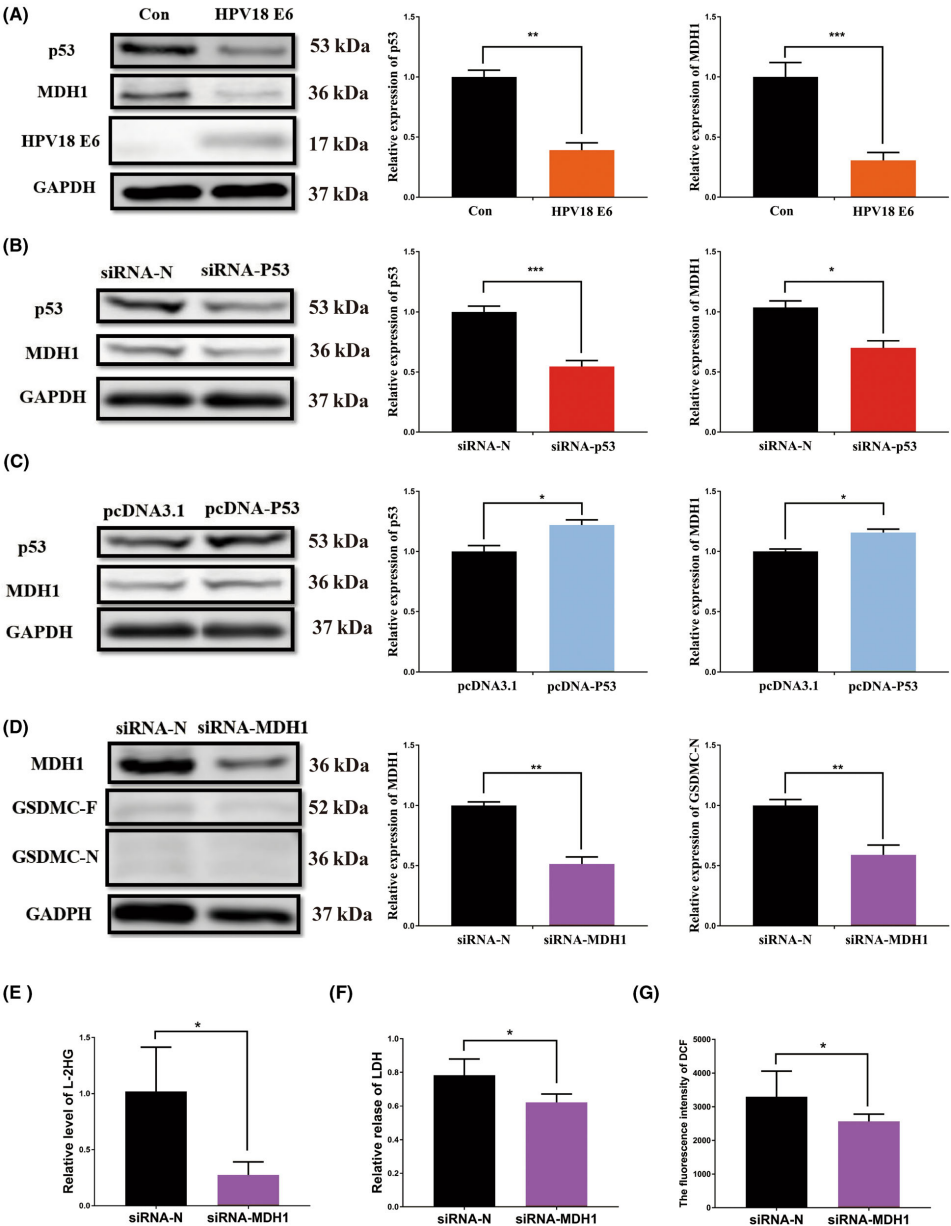
Human papillomavirus E6 inhibits pyroptosis via the p53/MDH1/ROS/GSDMC pathway. (A) Levels of p53 and MDH1 expression following HPV E6 expression detected via western blotting, data were analysed using Student's *t*‐test, **P < 0.01, ***P < 0.001. (B) Western blotting results for p53 and MDH1 levels detected after p53 knockdown, data were analysed using Student's *t*‐test, *P < 0.05, ***P < 0.001. (C) Western blot analysis results for p53 and MDH1 levels following overexpression of p53, data were analysed using Student's *t*‐test, *P < 0.05. (D) MDH1 and GSDMC expression levels detected via western blotting following MDH1 knockdown, data were analysed using Student's *t*‐test, **P < 0.01. (E) Relative levels of L‐2HG following MDH1 knockdown at a DM‐AKG concentration of 15 mm, data represent the mean ± SD of three independent experiments, data were analysed using Student's *t*‐test, *P < 0.05. (F) Relative levels of LDH released following MDH1 knockdown at a DM‐AKG concentration of 15 mm, data represent the mean ± SD of four independent experiments, data were analysed using Student's *t*‐test, *P < 0.05. (G) Levels of ROS produced following MDH1 knockdown at a DM‐AKG 15 mm concentration, data represent the mean ± SD of four independent experiments, data were analysed using Student's *t*‐test, *P < 0.05.

On further investigating how HPV E6 inhibits DM‐AKG induced pyroptosis, we found that the expression of p53 and MDH1 in ESCC decreases significantly following the HPV E6 expression (Fig. 4A). p53 is the direct target of HPV E6, and most of the carcinogenic functions of HPV E6 are realised via its influence on p53 [48, 49, 50, 51]. To investigate whether HPV E6 affects MDH1 via its impact on p53 expression, we overexpressed and then knocked down p53 in ESCC cells (Fig. S4B,C). The abnormal expression (i.e. overexpression) of p53 significantly impacted MDH1 expression and was positively correlated with MDH1 expression (Fig. 4B,C). Subsequently, we investigated whether MDH1 impacts pyroptosis. We found that MDH1 expression decreased, which inhibited the production of GSDMC‐N, L‐2HG and ROS and decreased the amount of LDH released (Fig. S4D, Fig. 4D–G). These results indicate that HPV E6 inhibits DM‐AKG‐induced pyroptosis of ESCC cells via the p53/MDH1/ROS/GSDMC pathway.

## Discussion

Pyroptosis is an inflammatory and programmed mode of cell death that is mediated primarily by members of the gasdermin family [2]. The relationship between members of the gasdermin family (including GSDMB, GSDMC, GSDMD and GSDME, but excluding GSDMA and PJVK) and pyroptosis and the related mechanism underpinning cutting into N terminus have been researched clearly understood [4]. Many drugs have been reported to induce pyroptosis, including granzyme A, lipopolysaccharide and Nigericin [9, 11].

Alpha‐ketoglutarate is a key metabolite in the TCA cycle and has considerable application significance. AKG has been used as a dietary supplement and plays a specific role in anti‐ageing and body improvement. It has been reported that AKG levels in the body increase with exercise and that AKG levels in the body are negatively correlated with body mass index (BMI). When BMI is as high as 20–25 and higher than 25, the levels of AKG in the body are significantly low [52, 53]. A considerable number of studies have demonstrated that exercise significantly improves the body function and survival rate of cancer patients, while obesity increases the incidence of various diseases [54, 55]. Exercise and obesity both determine the AKG levels in the body; hence, it is boldly speculated that AKG may play a positive role in the body developing and maintaining resistance to tumours and diseases. In an acidic environment, AKG can be catalysed by MDH1 to produce L‐2HG and ROS [27, 46, 47]. Due to the Warburg effect and mitochondrial dysfunction in cancer cells, lactate production in cancer cells is much higher than in normal cells, and TCA circulation levels are low [56]. It has been reported that AKG‐induced cytotoxicity is particularly significant in cells with mitochondrial dysfunction [25, 26]. Therefore, we surmise that following the addition of AKG, the main metabolic pathway of AKG is blocked due to the low TCA circulation levels and high aerobic glycolysis levels of tumour cells, which engenders the conversion of AKG into L‐2HG, which releases ROS and thus produces cytotoxicity. By screening for the toxic effects of AKG on five kinds of ESCC cells and three kinds of cervical cancer cells, we found that a high dose of AKG has a significant toxic effect on these cells and exhibits characteristics of the phenomenon of pyroptosis (Fig. S1). Subsequently, we confirmed that AKG‐induced pyroptosis is caused by ROS produced during the conversion of AKG to L‐2HG, not by L‐2HG. Furthermore, the AKG‐induced pyroptosis is executed via the GSDMC pathway, not GSDMB, GSDMD or GSDME. However, it has been reported that ROS produced by AKG can induce pyroptosis, and ROS can induce the internalisation of DR6, thereby recruiting caspase‐8 to shear GSDMC [27].

It has been reported that high concentrations of succinic acid can induce the apoptosis of renal cell carcinoma and endometrial carcinoma cells [57]. AKG and succinic acid are both key products in the TCA cycle. High concentrations of AKG can also induce tumour cell death; hence, we believe that AKG has great prospects and potential applications in antitumour research. Exploiting the metabolic differences between tumour cells and normal cells, identifying several metabolic intermediates for research and drug development may be a feasible approach to tumour treatment.

Human papillomavirus is a double‐stranded DNA virus that readily infects mucosal epithelial tissues in the anal genitalia and the upper respiratory and digestive tracts [32]. HPV is the primary carcinogenic factor in cervical cancer and is closely linked to the occurrence and development of esophageal cancer, vaginal cancer, anal cancer, and head and neck cancer [32, 33]. Several studies have shown that most esophageal cancer specimens also have an HPV infection and that an HPV infection is also a negative prognostic factor in esophageal cancer patients [30, 31]. In addition, HPV has been shown to inhibit a variety of cell death modes, including cell necrosis, apoptosis and autophagy [36, 37, 38, 39]. However, the relationship between HPV and pyroptosis has been studied by only a few researchers. We found that HPV‐positive tumour cells have a high survival rate under a high dose of AKG and significantly inhibit AKG‐induced pyroptosis. To determine the role of HPV E6 and HPV E7, we constructed HPV E6‐ and HPV E7‐positive cells in HPV‐negative KYSE150 cells to verify the roles of E6 and E7 in AKG‐induced pyroptosis inhibited by HPV. Subsequently, we confirmed that HPV E6 significantly inhibits AKG‐induced pyroptosis of ESCC cells. Because p53 is the most important target of HPV E6, we found that MDH1 expression is positively correlated with p53 expression by knocking down and overexpressing p53. Furthermore, a decrease in MDH1 expression levels significantly inhibited the production of L‐2HG, ROS and GSDMC‐N. p53 is the main target of HPV E6—this has been confirmed and is generally recognised. Whether the relationship between p53 and MDH1 is direct or indirect is unknown, and there is a dearth of relevant studies on this subject; hence, further research is needed. Finally, in addition to the results reported in this study, we propose that HPV E6 inhibits AKG‐induced pyroptosis of ESCC via the p53/MHD1/ROS/GSDMC pathway.

## Conclusions

We found that AKG can have a toxic effect on various tumour cells. Through an in‐depth study of ESCC cells, we found that AKG‐induced cell death exhibits the characteristics of pyroptosis, including GSDMC‐induced pyroptosis. AKG‐induced pyroptosis is caused by ROS produced during the conversion of AKG to L‐2HG. The presence of HPV significantly inhibits AKG‐induced pyroptosis, which is primarily attributable to HPV E6. After knocking down and overexpressing the p53 protein, we found that MDH1 expression is positively correlated with p53 expression. However, low MDH1 expression downregulates the production of L‐2HG, ROS and GSDMC‐N. Finally, we propose that HPV E6 inhibits AKG‐induced pyroptosis of ESCC cells via the p53/MHD1/ROS/GSDMC pathway.

## Conflict of interest

The authors declare no conflict of interest.

### Peer review

The peer review history for this article is available at https://www.webofscience.com/api/gateway/wos/peer‐review/10.1002/2211‐5463.13666.

## Author contributions

ZZ and DT designed the study and performed the experiments. ZL, GW, BW and QZ performed the experiments and data analysis, and made considerable contributions to the drafting of the manuscript. Y Zheng, XH, Y Zhang, MY and CS were responsible for the collection and analysis of case data and literature. All authors edited the manuscript. All authors read and approved the final manuscript.

## Supporting information


**Fig. S1.** HPV18 E6 and E7 sequence optimisation. (A) HPV18 E6 sequence optimisation. (B) HPV18 E7 sequence optimisation.

**Fig. S2.** Toxic effects of AKG. (A) Toxic effects of DM‐AKG on KYSE150, KYSE180, KYSE450, EC109, EC9706, C33a, Siha, and Hela cells, data represent the mean ± SD of three independent experiments. (B) A 15 mM DM‐AKG concentration significantly promoted cell death, data represent the mean ± SD of three independent experiments, data were analysed using Student's t‐test, * p < 0.05.

**Fig. S3.** Survival rate after exposure to DM modified compounds after 24 h. (A) Survival rate after exposure to DM‐AKG and DM‐Succinate, data represent the mean ± SD of three independent experiments. (B) Survival rate after exposure to 15 mM DM‐AKG and DM‐Succinate, data represent the mean ± SD of three independent experiments, data were analysed using one‐way ANOVA with Tukey's *post hoc* test, *** p < 0.001. (C) Survival rate after exposure to 30 mM DM‐AKG and DM‐Succinate, data represent the mean ± SD of three independent experiments, data were analysed using one‐way ANOVA with Tukey's *post hoc* test, *** p < 0.001.

**Fig. S4.** Relative transcript level of GSDMC, P53 and MDH1. (A) The relative transcript level of GSDMC, data represent the mean ± SD of four independent experiments, data were analysed using Student's t‐test, *** p < 0.001. (B & C). The relative transcript level of P53, data represent the mean ± SD of four independent experiments, data were analysed using Student's t‐test, * p < 0.05, ** p < 0.01. (D) The relative transcript level of MDH1, data represent the mean ± SD of four independent experiments, data were analysed using Student's t‐test, *** p < 0.001.

**Fig. S5.** HPV E6 inhibited a toxic effect induced by DM‐AKG. (A) Comparison of the toxic effect of DM‐AKG on KYSE150 and KYSE150‐E6 cells, data represent the mean ± SD of three independent experiments, data were analysed using Student's t‐test, * p < 0.05. (B) Comparison of the toxic effect of DM‐AKG on C33A and C33A‐E6 cells, data represent the mean ± SD of three independent experiments, data were analysed using Student's t‐test, ** p < 0.01.

**Movie S1.** AKG‐mediated pyrolysis of KYSE150 cells.

## Data Availability

The data presented in this study are contained within the article or [Link], [Link], [Link], [Link], [Link], [Link].The data presented in this study are available from the corresponding author upon reasonable request.
