# Supplementary figures and images for: HPV18 E6 inhibits α‐ketoglutarate‐induced pyroptosis of esophageal squamous cell carcinoma cells via the P53/MDH1/ROS/GSDMC pathway
Source: FEBS Open Bio. 2023 Jul 8;13(8):1522–35. doi: 10.1002/2211-5463.13666 (PMC10392054; doi:10.1002/2211-5463.13666)

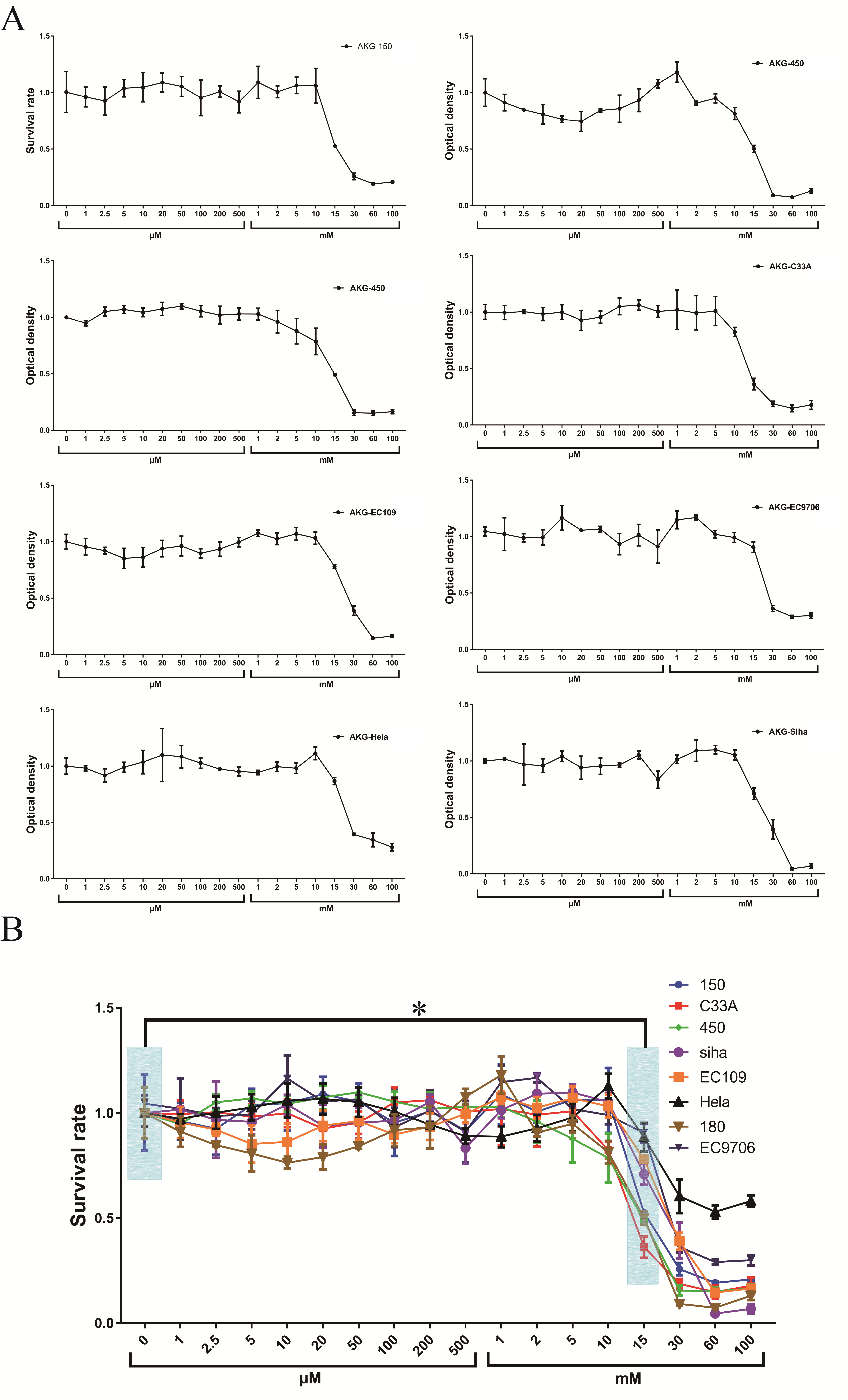

Supplement: Supplementary file 2 — Fig. S2. Toxic effects of AKG. (A) Toxic effects of DM‐AKG on KYSE150, KYSE180, KYSE450, EC109, EC9706, C33a, Siha, and Hela cells, data represent the mean ± SD of three independent experiments. (B) A 15 mM DM‐AKG concentration significantly promoted cell death, data represent the mean ± SD of three independent experiments, data were analysed using Student's t‐test, * p < 0.05. [file FEB4-13-1522-s006.tif]

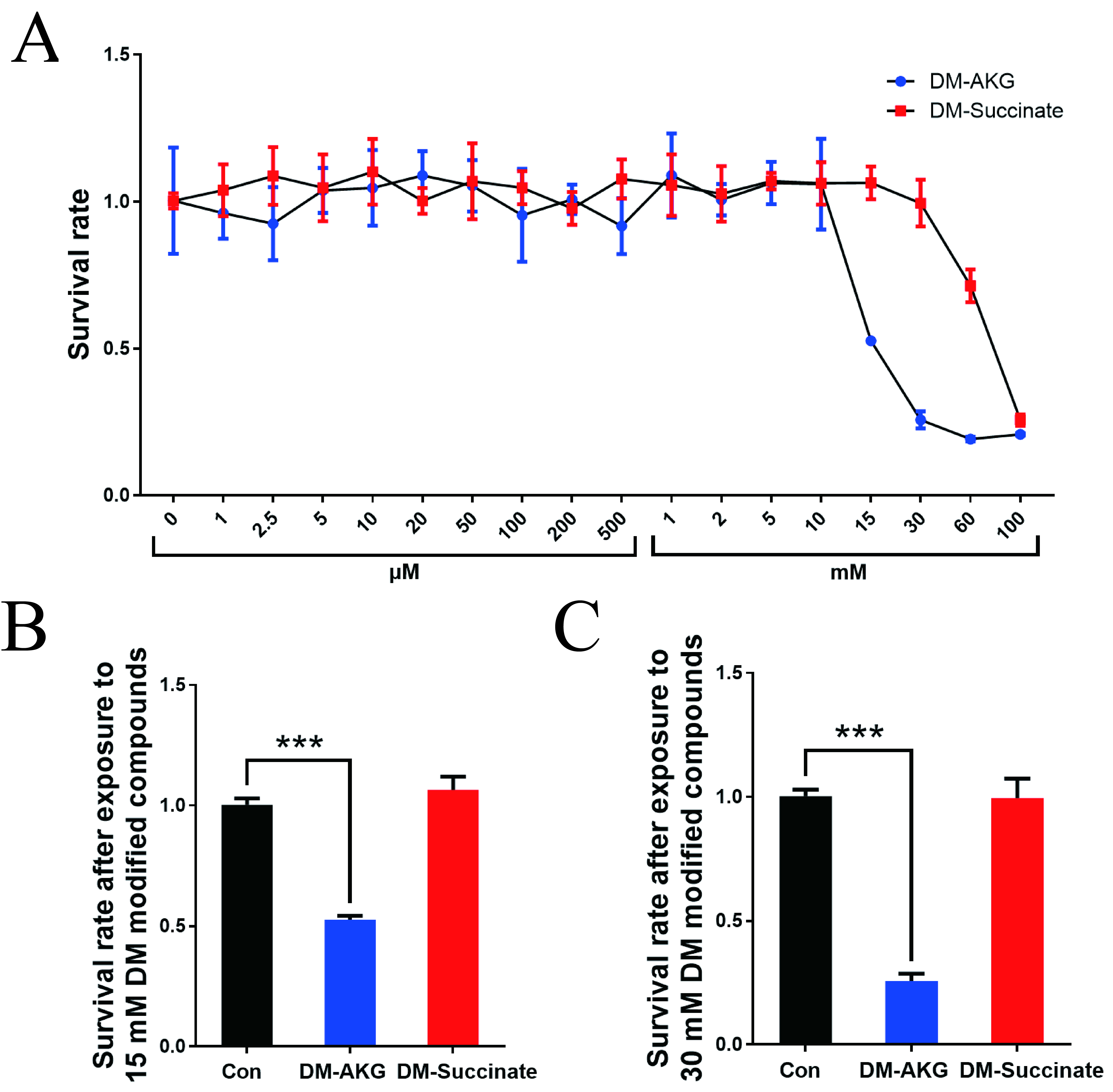

Supplement: Supplementary file 3 — Fig. S3. Survival rate after exposure to DM modified compounds after 24 h. (A) Survival rate after exposure to DM‐AKG and DM‐Succinate, data represent the mean ± SD of three independent experiments. (B) Survival rate after exposure to 15 mM DM‐AKG and DM‐Succinate, data represent the mean ± SD of three independent experiments, data were analysed using one‐way ANOVA with Tukey's post hoc test, *** p < 0.001. (C) Survival rate after exposure to 30 mM DM‐AKG and DM‐Succinate, data represent the mean ± SD of three independent experiments, data were analysed using one‐way ANOVA with Tukey's post hoc test, *** p < 0.001. [file FEB4-13-1522-s005.tif]

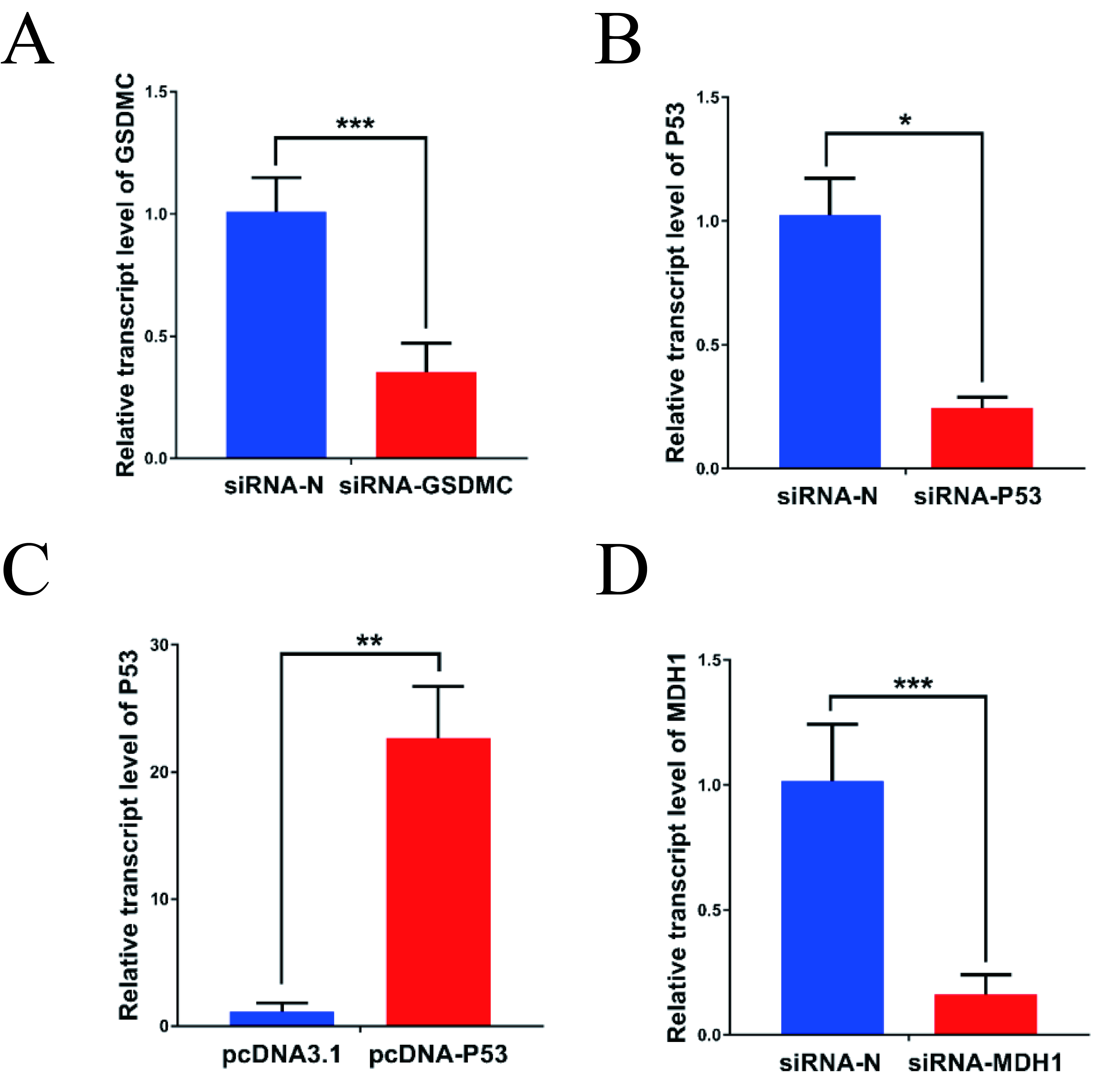

Supplement: Supplementary file 4 — Fig. S4. Relative transcript level of GSDMC, P53 and MDH1. (A) The relative transcript level of GSDMC, data represent the mean ± SD of four independent experiments, data were analysed using Student's t‐test, *** p < 0.001. (B & C). The relative transcript level of P53, data represent the mean ± SD of four independent experiments, data were analysed using Student's t‐test, * p < 0.05, ** p < 0.01. (D) The relative transcript level of MDH1, data represent the mean ± SD of four independent experiments, data were analysed using Student's t‐test, *** p < 0.001. [file FEB4-13-1522-s001.tif]

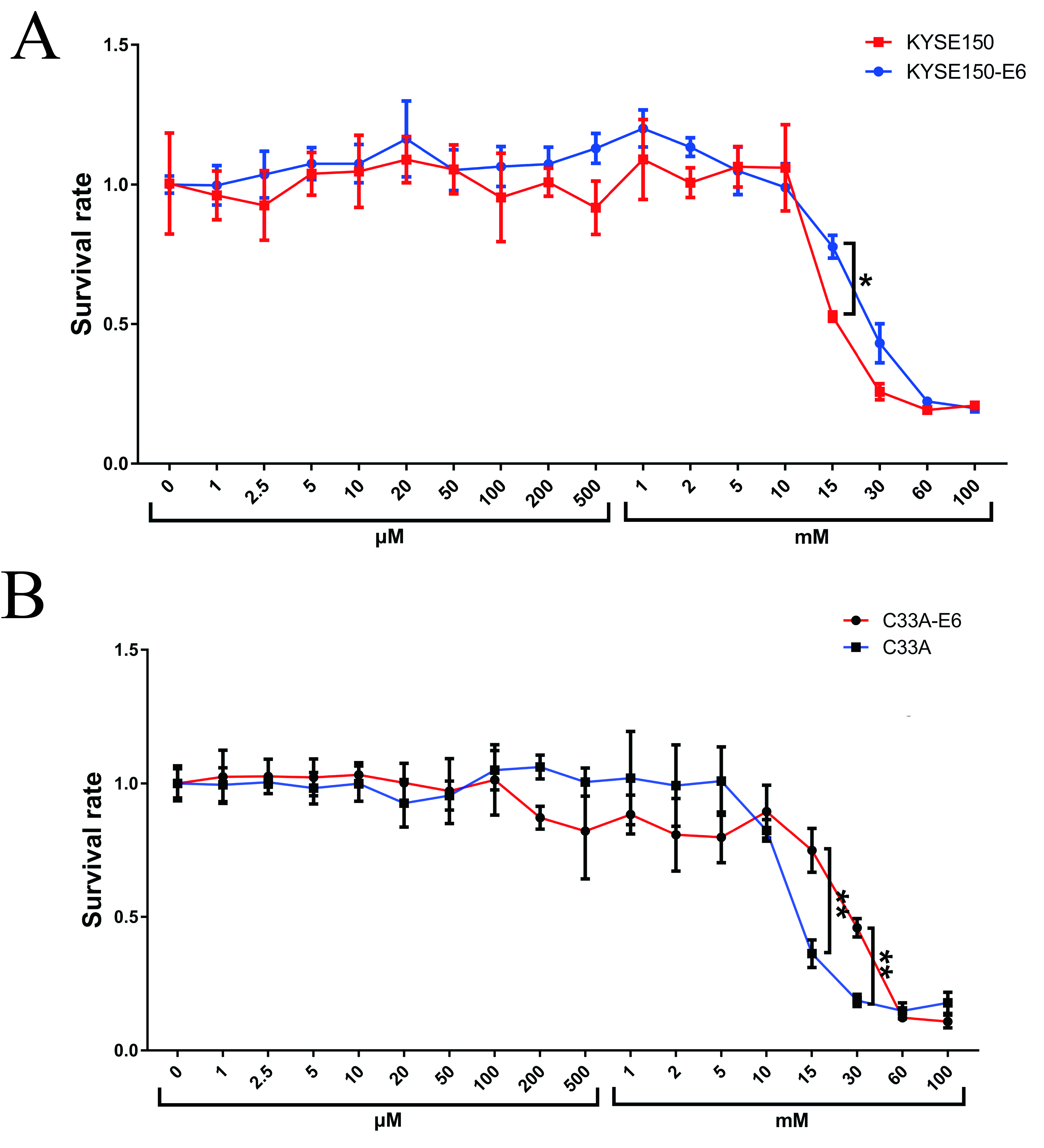

Supplement: Supplementary file 5 — Fig. S5. HPV E6 inhibited a toxic effect induced by DM‐AKG. (A) Comparison of the toxic effect of DM‐AKG on KYSE150 and KYSE150‐E6 cells, data represent the mean ± SD of three independent experiments, data were analysed using Student's t‐test, * p < 0.05. (B) Comparison of the toxic effect of DM‐AKG on C33A and C33A‐E6 cells, data represent the mean ± SD of three independent experiments, data were analysed using Student's t‐test, ** p < 0.01. [file FEB4-13-1522-s002.tif]
